# Bioherbicidal Activity of *Albifimbria verrucaria* (Formerly *Myrothecium verrucaria*) on Glyphosate-Resistant *Conyza canadensis*

**DOI:** 10.3390/jof9070773

**Published:** 2023-07-22

**Authors:** Robert E. Hoagland, C. Douglas Boyette, Kenneth C. Stetina

**Affiliations:** 1 USDA-ARS, Crop Production Systems Research Unit, Stoneville, MS 38776, USA; 2 USDA-ARS, Biological Control of Pests Research Unit, Stoneville, MS 38776, USA

**Keywords:** *Albifimbria verrucaria*, *Myrothecium verrucaria*, *Conyza canadensis*, horseweed, bioherbicide, biological weed control

## Abstract

The effects of the bioherbicidal activity of the fungal phytopathogen, *Albifimbria verrucaria* (AV), formerly *Myrothecium verrucaria*, on glyphosate-resistant and –susceptible *Conyza canadensis* (horseweed) were examined in greenhouse and field studies. Spray applications of mycelial formulations of AV infected both glyphosate-resistant and -susceptible *C. canadensis* plants at various growth stages. Young plants in the rosette leaf stage of growth were controlled more efficaciously than were older plants that had bolted or that were in the inflorescence stage; nevertheless, severe injury and mortality also occurred in mature plants. The results indicate that this bioherbicidal fungus can infect and control *C*. *canadensis*, thereby demonstrating the potential of this fungus as a bioherbicidal agent against this troublesome weed, which has become resistant to various herbicides.

## 1. Introduction

Horseweed (*Conyza canadensis* L. Cronq.), also called *Erigeron canadensis* L., marestail, colt’s tail, and Canada fleabane, is an annual weed belonging to the Asteraceae family and is native to, and commonly distributed throughout, North America [1]. It has been reported to infest numerous crops in 70 countries [1,2] and is troublesome in several major crops, including cotton [3], grain sorghum [4], corn [5,6], and soybean [6] in the U.S. It can also be problematic in fallow, in abandoned and in reduced tillage fields [7,8]. Commercial nurseries experience disruption caused by the weed during the culture of ornamentals [9]. Horseweed plants serve as hosts for the tarnished plant bug (*Lygus lineolaris*), which can plague various agricultural crops [10], as well as a viral disease (aster yellows) that is transported and transmitted to other plants via leafhoppers (*Macrosteles fascifrons* (Stal)) [1,10,11].

A single plant may produce ~200,000 seeds that are highly mobile and dispersible via the wind [12,13]. Horseweed is tolerant to many herbicides [14] and can establish under a wide range of soil types and environmental conditions [13,15]. It can emerge after corn planting [5] and through the spring season in no-tillage fields [12]. The commonly used post-emergence herbicides for soybean do not provide control of established horseweed [16].

Horseweed’s occurrence can increase under reduced tillage and no-till situations [17]. The weed not only reduces crop yields via direct competition, it contains allelochemicals reported to inhibit the germination and seedling growth of several plant species [18]. Horseweed tissues also contain compounds that are irritating to the nostrils of horses [19].

Aside from being a widespread weed problem, some *C. canadensis* biotypes have evolved resistance to one or more herbicides [20]. The first report of herbicide resistance in *C. canadensis* was resistance to paraquat in 1980 [20]. Since then, other populations of horseweed plants with resistance to paraquat [21,22], triazine herbicides [23], and 12 other herbicides [20] have been documented. The herbicide glyphosate initially provided a high level of control of *C. canadensis* [14,24,25], although in the year 2000, glyphosate-resistant *C. canadensis* was first reported in Delaware, USA [26], and now occurs in 14 countries and in over 2 dozen U.S. states [20]. Some of these glyphosate-resistant biotypes are also resistant to other herbicides [20]. The acceptable control of horseweed has been achieved with tank mixtures of glyphosate with saflufenacil, saflufenacil/dimethenamid-P, metribuzin, cloransulam, or flumetsulam applied pre-emergence [27]. The relatively new herbicide tiafenacil provided acceptable control of glyphosate-resistant horseweed in corn when combined with some other herbicides, although the control was significantly lower when the compound was applied alone [28]. Mechanical weed management can be useful for control. However, since the early stages of horseweed establishment are extremely sensitive to dehydration, shallow tillage should be accomplished soon after a rain event or in the fall and spring to increase the probability of dehydration and death of young seedlings [29].

Due to the problems of controlling horseweed and its wide distribution and resistance to many herbicides, other strategies of control may need to be considered. The topic of using microbial bioherbicides (fungi and bacteria) for controlling weeds and as potential alternatives to synthetic herbicides has been a subject of study for several decades, as summarized in selected books and reviews [30,31,32,33,34]. This bioherbicide concept is fueled in part by the increasing number of weeds resistant to synthetic herbicides [20], a lack of new commercial herbicides with novel modes of action, and environmental goals to use less chemicals or to develop non-chemical or bioherbicidal weed management strategies, as outlined in selected reviews [35,36,37,38,39,40].

Various bioherbicidal factors (virulence, host range, and epidemiology) of a fungal phytopathogen, *Myrothecium verrucaria* (isolate IMI 368023), have been accessed in studies on various plants and weeds [41,42,43]. Recent taxonomic studies resulted in the reclassification of this *Myrothecium verrucaria* isolate as *Albifimbria verrucaria* (AV) [44]. Prior to this taxonomic reclassification, this fungus was shown to exhibit high bioherbicidal activity on several weeds including kudzu [45], morning glory [46], hemp sesbania [47], and glyphosate-resistant Palmer amaranth [48]. In other recent studies, we found preliminary evidence (unpublished) that AV could injure *C. canadensis* seedlings under environmental chamber conditions. The objectives of the current studies were to expand these preliminary findings and to determine the bioherbicidal efficacy of AV based on the plant growth stage of both glyphosate-resistant and -susceptible *C. canadensis* seedlings under greenhouse and field conditions. Knowledge of these epidemiological parameters is critical in order to evaluate the performance of candidate bioherbicides as weed control agents and is especially important when considering the control of herbicide resistant weeds.

## 2. Materials and Methods

### 2.1. Seed Collection and Plant Culture

*C. canadensis* seeds were collected from various field sites near USDA-ARS, Stoneville, MS. The seeds were germinated on dampened filter paper in an environmental chamber (100% relative humidity (RH)) for 48 h, then planted in a 2:1 mixture of potting soil mix and sandy soil contained in 10 cm^2^ pots and grown under greenhouse conditions (28–32 °C, 40–60% RH, ~14 h day, at 1650 µEm^−2^s^−1^ photosynthetically active radiation (PAR), measured at midday). De-ionized water was supplied to the plants daily and dilute fertilizer (N/P/K (13:13:13)) was provided biweekly. When the plants reached the rosette growth stage, leaf disks were cut from the excised leaves and bio-assayed to assess variations in susceptibility or tolerance to the technical grade (98% pure), as described elsewhere [49]. The plants were then divided into two groups, glyphosate-susceptible (GS) and glyphosate-resistant (GR), and were grown under the same conditions and tested at several growth stages for effects caused by the fungal bioherbicide AV. Specifically, the growth stages tested were the 5 to 8 rosette leaf stage, 11 to 15 rosette leaf stage, bolting (flowering) stages, 5 to 15 cm tall and 16 to 90 cm tall in greenhouse tests, and bolting stages of 55 to 60 cm tall in the field tests.

### 2.2. AV Source and Production

Cultures of AV (formerly *M. verrucaria* (IMI 368023)) were maintained on potato dextrose agar (PDA) (Difco Laboratories, Inc., Detroit, MI, USA) in petri dishes at 25 °C. Mycelial cultures of AV were prepared via fermentation and used as previously described [50,51].

### 2.3. Spray Application of AV to Greenhouse and Field Plants

The freshly prepared mycelial product was homogenized (electric blender, 60 s, high speed) and used directly for experimentation in greenhouse and field tests. A freshly prepared mycelial formulation containing the surfactant (Silwet-L77™; OSi Specialties, Inc., Danbury, CT, USA) at 0.20% (*v*/*v*) was sprayed onto *C. canadensis* plants until the leaves and tissues were wet (ca. 200 L/ha) using a hand-pump sprayer (Spray-Doc, Gilmor Multi-Purpose Sprayer, BFG Supply Co., Burton, OH, USA). The control plants received spray applications of 0.20% surfactant in deionized water. The plant pots were then placed on trays on greenhouse benches and monitored over a 7-day period for disease development. In the field tests, uniform plants were randomly selected (50–60 cm tall) in naturally infested areas and used for spray applications of the pathogen. The control and inoculated field plants were treated as described above for the greenhouse tests and the plant and disease symptomology was monitored over a 7-day period. A modified Horsfall–Barrett disease rating scale [52] was used as a visual disease severity rating scale to estimate disease progression or severity. The linear scale ratings were defined as follows: 0 = no disease symptoms; 1 = 10%, 3 = 30%; 5 = 50%; 8 = 80% disease injury of leaves and stems; 10 = 100% injury or plant mortality.

### 2.4. Statistics

All greenhouse experiments were conducted in triplicate for each treatment and each experiment was arranged in a randomized complete block design. All experiments were repeated. The field experiments were also arranged in a completely randomized design with 6 to 8 plants per replication and the experiment was repeated. The means were pooled and subjected to an analysis of variance (Fisher’s LSD_0.05_ or S.E.M. analysis) using SAS (version 9.1, SAS Institute, Inc., Cary, NC, USA) statistical software. Because the greenhouse data from the glyphosate-resistant versus –susceptible plants were not significantly different, only data from the glyphosate-resistant plants are displayed. The plants used in field tests were not tested for resistance or susceptibility to glyphosate, although since the data from the greenhouse tests demonstrated no differences of these pathogens’ effects on glyphosate-resistant or -susceptible plants, it is assumed that the AV effects are identical regardless of the degree of glyphosate tolerance of individual plants. The percentage of weed control was calculated by dividing the number of severely injured plants + dead plants (symptom ratings of 8.0 to 10.0) by the total number of plants inoculated × 100. Standard mean errors and a best-fit regression analysis were accomplished using SAS as indicated above.

## 3. Results

### 3.1. Greenhouse Tests

**Figure 1:**
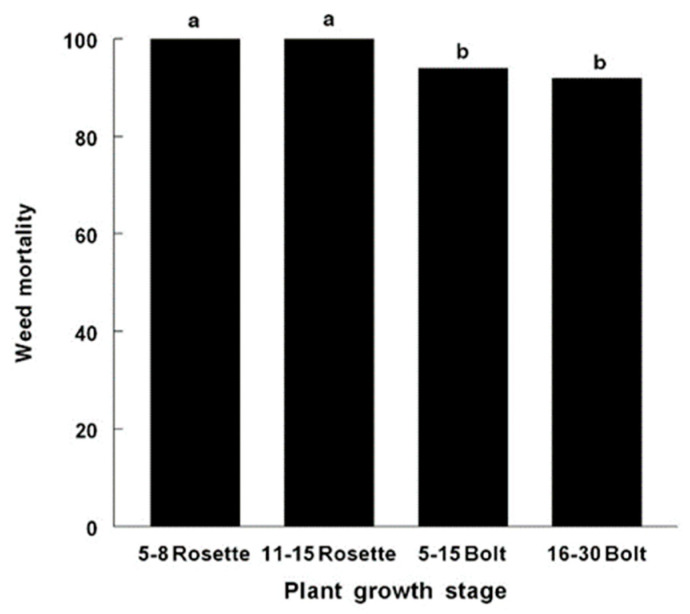
Effects of AV on C. canadensis at various growth stages under greenhouse conditions. The inoculum concentration was a full-strength mycelium product produced from a fermenter as described in the Materials and Methods. Since there were no significant differences (Fishers LSD0.05) in mortality at any plant growth stage, statistical symbols are not shown. Specific differences between pairs of means were analyzed. Mean values of histogram bars with the same letter are not statistically different at *p* = 0.05.

**Figure 2:**
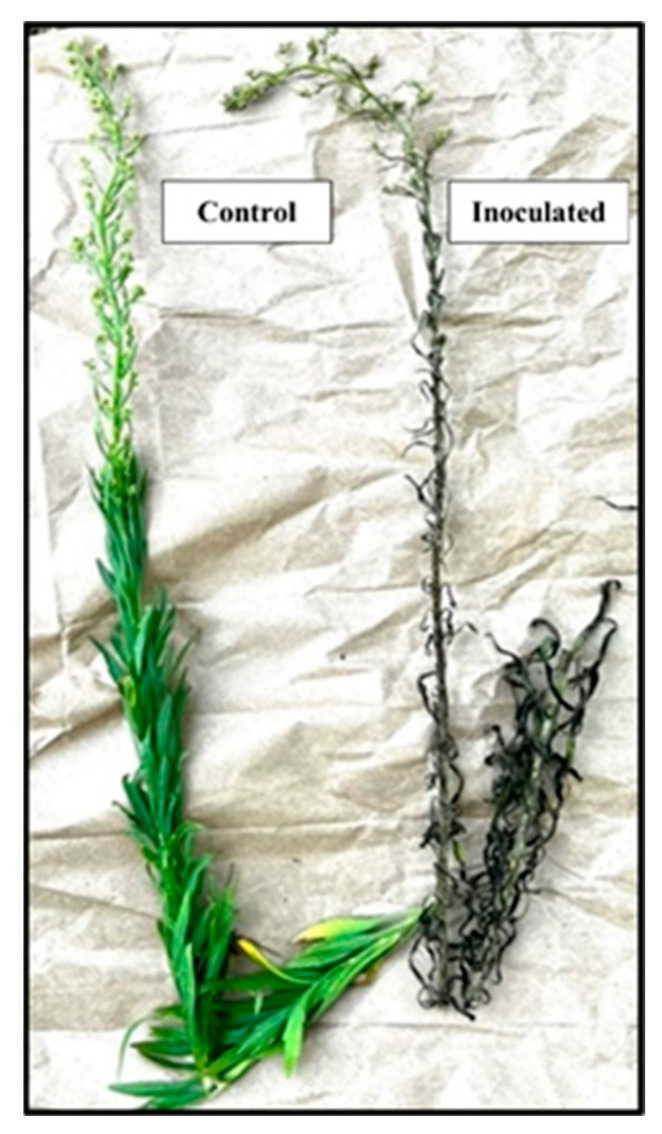
Effect of AV on *C. canadensis* at the inflorescence growth stage under greenhouse conditions 46 h after inoculation. Plant heights ranged from 56 to 60 cm.

**Figure 3:**
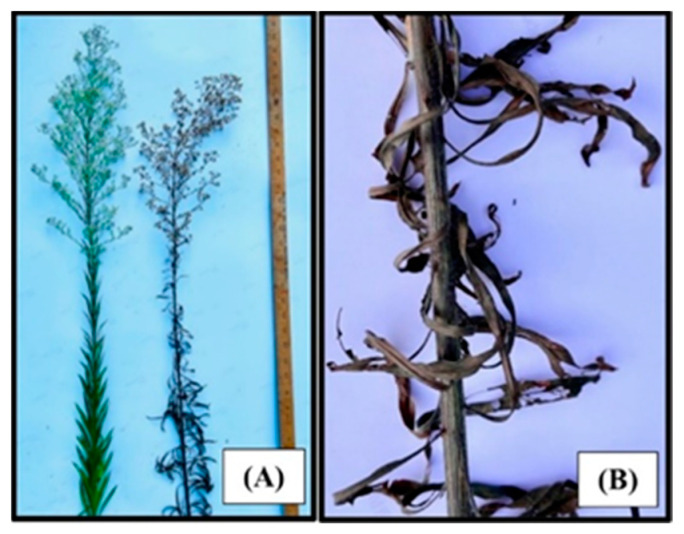
Effect of AV on *C. canadensis* at the inflorescence growth stage under greenhouse conditions 6 days after inoculation: (**A**) a typical plant from this group, which ranged 85–90 cm; (**B**) a closeup view of a necrotic stem and leaves from the AV-inoculated plant.

The high mortality (%) of *C. canadensis* was caused by AV in plants in the rosette and bolting growth stages, 7 days after inoculation under greenhouse conditions without subjecting the inoculated plant to an exposure period of supplemental moisture (dew) (Figure 1). No significant differences were found in the glyphosate-susceptible versus glyphosate-resistant plants; therefore, only data from glyphosate-resistant plants are presented. Intermediate-sized horseweed plants (56–60 cm tall) in the early inflorescence stage were severely injured by the AV inoculum treatment. Plants at this growth stage and size exhibited severe necrosis, especially on the leaves and stems at 46 h after inoculation (Figure 2). Tall plants in the late inflorescence stage (85–90 cm tall) were killed at 6–7 days after inoculation under greenhouse conditions (Figure 3A). The leaves and stems were totally necrotic, as shown in a close-up photo (Figure 3B).

### 3.2. Field Tests

**Figure 4:**
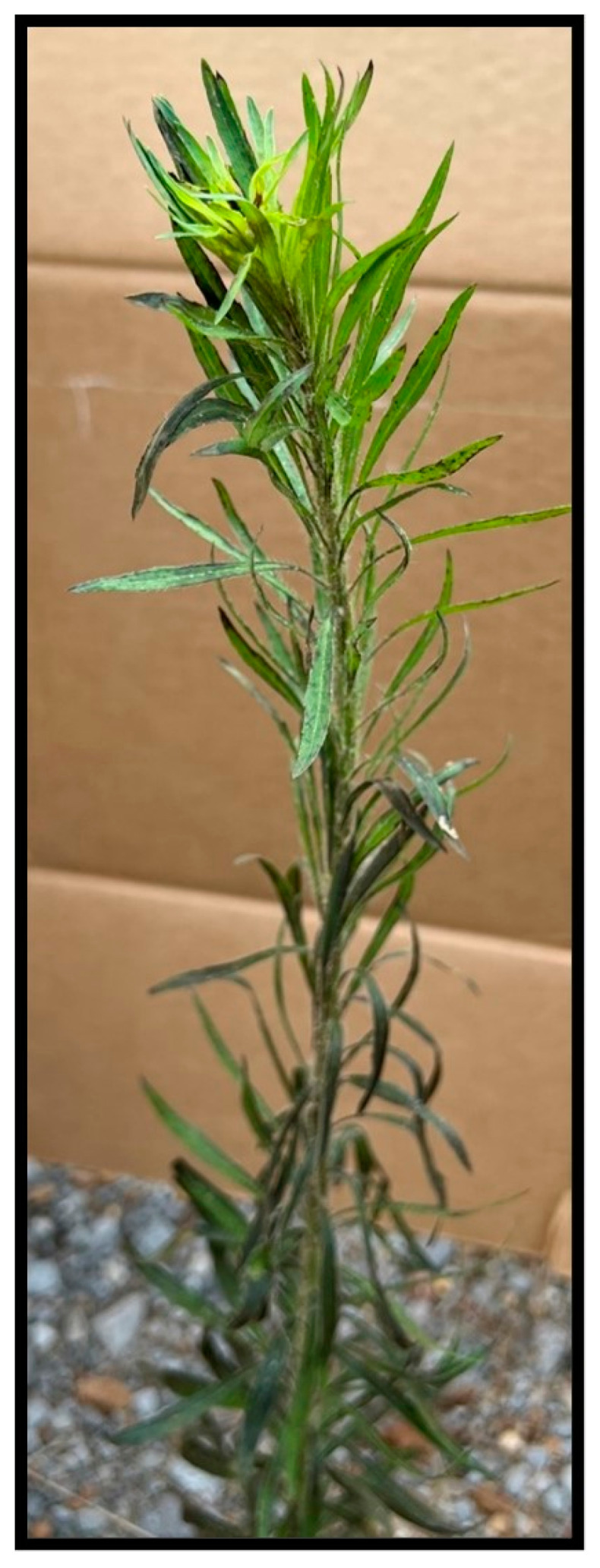
Effect of AV on *C. canadensis* inoculated at the pre-inflorescence growth stage under field conditions, 48 h after inoculation. Plants ranged 50–55 cm in height at the time of inoculation. Note some necrotic leaves and chlorosis occurring in meristematic leaves.

**Figure 5:**
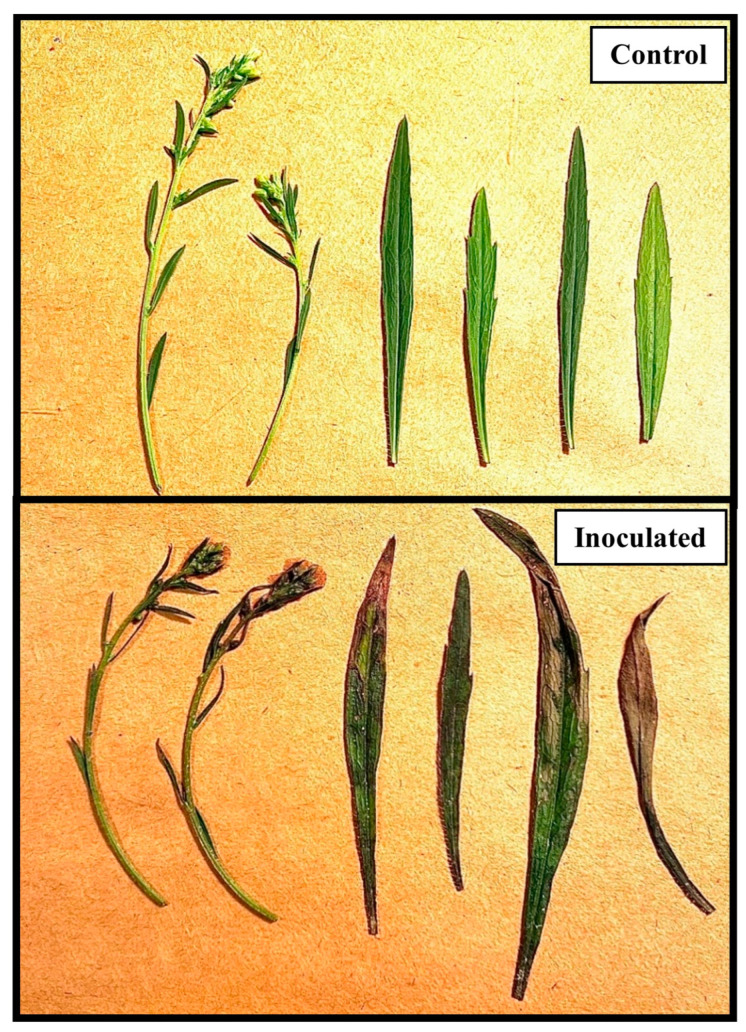
Excised plant parts showing the effects of AV on *C. canadensis* inoculated at the inflorescence growth stage under field conditions, 40 h after inoculation. Plants ranged 55–60 cm tall at the time of inoculation.

**Figure 6:**
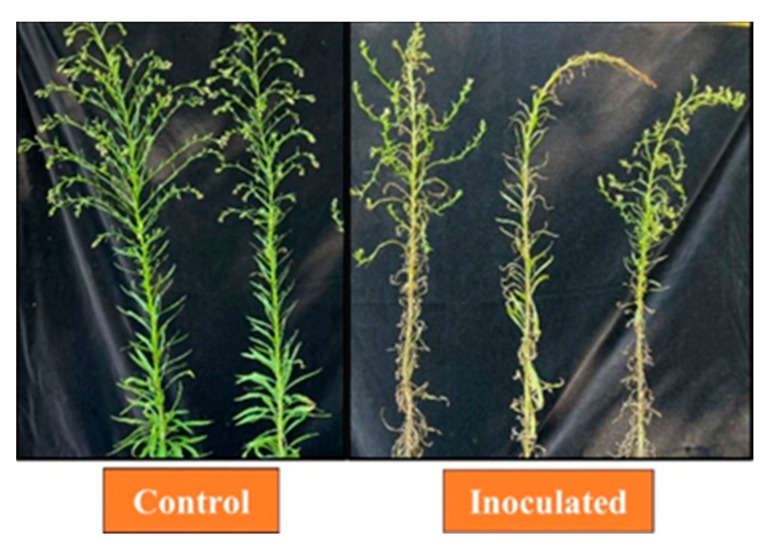
Effect of AV on *C. canadensis* inoculated at the inflorescence growth stage under field conditions, 55 h after inoculation. Plants ranged 55–60 cm in height at the time of inoculation.

The tests of AV efficacy on horseweed plants of varying sizes and developmental stages were also carried out on field plants in natural settings. Intermediate-sized horseweed plants (50–55 cm tall) in the pre-inflorescence stage exhibited some bioherbicidal effects such as leaf necrosis (especially in lower leaves) and chlorosis, mostly localized in the meristematic leaves, 48 h after inoculation under field conditions (Figure 4). The injury to the plants increased with time, and at 40 h after inoculation, some necrotic leaves detached from the stems, while the others exhibited varying degrees of necrosis, chlorosis, and death or mortality compared to tissues from un-inoculated (control) plants (Figure 5). When the AV spray inoculations were applied to *C. canadensis* at the inflorescence growth stage under field conditions, the growth was significantly reduced, some necrotic dead leaves were sluffed-off, and the inflorescence plant parts (branches and flowers) and plant stems exhibited major bioherbicidal injury 55 h after inoculation (Figure 6). The plants height ranged 55–60 cm at the time of inoculation. All tests under field conditions were performed at temperature ranges of 32–36 °C (highs) and 20–23 °C (lows). Occasional minor rain events occurred (<0.20 cm).

### 3.3. AV Disease Progression on Greenhouse and Field Plants

**Figure 7:**
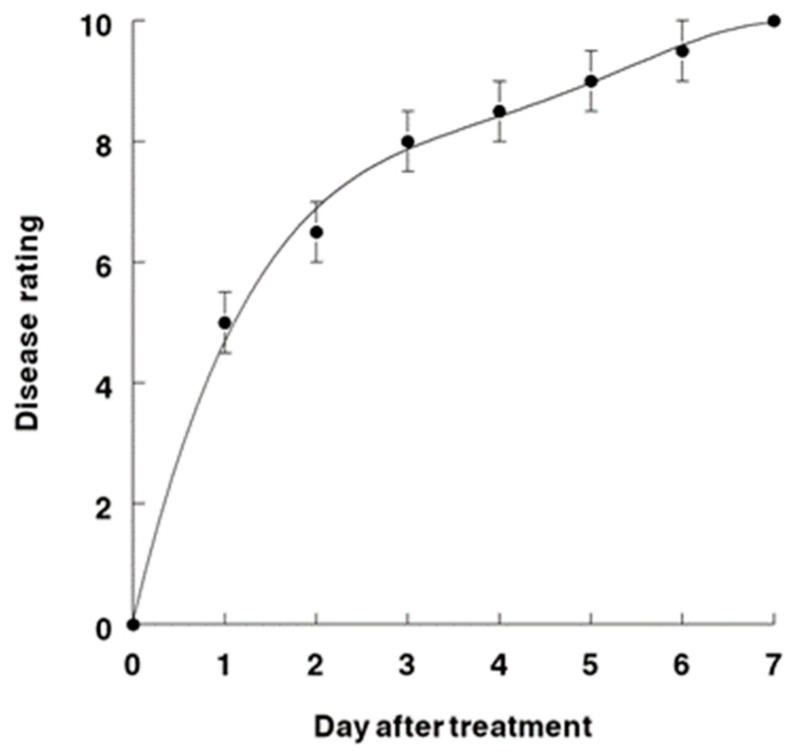
AV disease severity on *C. canadensis* over a 7-day time course under greenhouse conditions. This curve is best represented by a fourth-degree polynomial regression curve: Y= 0.08 + 6.31X − 1.97X2 + 0.29X3 − 0.02 X4; R^2^ = 0.98.

**Figure 8:**
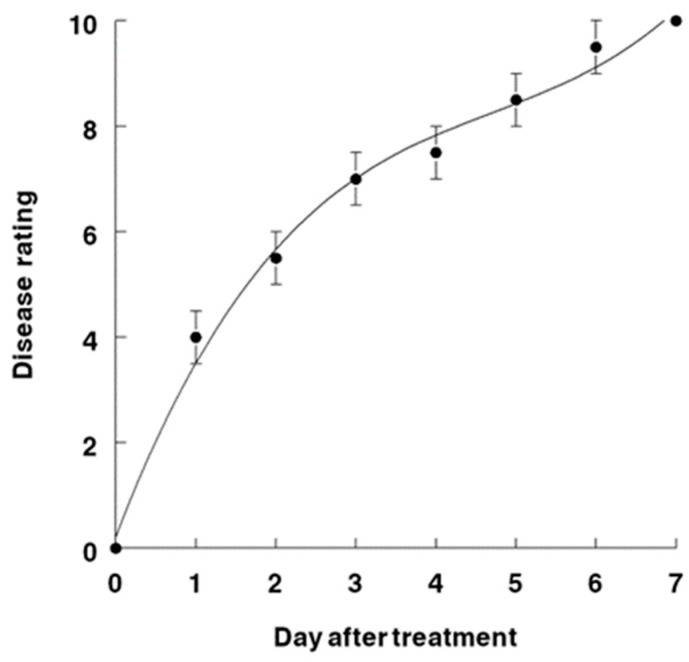
AV disease severity on *C. canadensis* over a 7-dat time course under field conditions. This curve is best represented by a third-degree polynomial regression curve: Y= 0.22 + 3.95X − 0.71X2 + 0.05X3; R^2^ = 0.98.

The disease progression of AV on horseweed was compared in tests on greenhouse-grown versus field-grown plants of equivalent sizes (55–60 cm tall) (Figure 7 and Figure 8). The disease symptomology progressed slightly more rapidly under controlled and more moderate conditions of the greenhouse, i.e., after 24 h, the disease rating was about 5.0 versus 4.0 for the greenhouse versus field plants, respectively. This trend continued, and at 4 days after inoculation, the ratings were 8.2 (greenhouse) versus 7.2 (field). However, at 7 days after inoculation, both the greenhouse and field plants exhibited essentially the same rating of 10.0, which indicates severe injury or plant mortality. The disease progression curve of AV disease severity on inoculated horseweed plants over a 7-day time course under greenhouse conditions was best represented by a fourth-degree polynomial regression curve, where R^2^ = 0.98 (Figure 7). Similarly, a third-degree polynomial regression curve (R^2^ = 0.98) was the best fit for the disease progression of AV on horseweed in field tests (Figure 8).

## 4. Discussion

Most of the microorganisms studied for bioherbicidal activity have been fungi, with few bacterial phytopathogens evaluated for weed control. This point also holds true for the bioherbicidal studies conducted in our laboratory. However, regarding horseweed, we found a bacterial pathogen (*X. campestris*) that was an effective biocontrol agent, although a requirement of a relatively long (20 h) free-moisture (dew) period following inoculation was needed to achieve weed control levels of ~80% mortality [53]. In contrast, a dew duration period of only 16 h resulted in relatively high (80%) control of cocklebur (*Xanthium strumarium*) plants treated with another *Xanthomonas* strain [54,55]. No plant mortality occurred at ≤4 h of dew [51] and mature plants were more resistant to infection than younger plants [55]. In the present studies, no dew was applied in the greenhouse tests, although plants in the field test did receive intermittent dew and some light rain (<0.20 cm) during testing. Dew is generally thought to be advantageous to promote the disease development of bioherbicides. However, although rainfall provides moisture, it could also cause the wash-off of pathogen propagules (spores or mycelial fragments). The lack of a dew requirement for AV to achieve a relatively high degree of control on horseweed is an important finding. In another study, the effects of simulated rainfall and dew on disease development and weed control using *Alternaria cassiae* and *Colletotrichum truncatum*, bioherbicidal pathogens of sicklepod (*Senna obtusifolia* (L.) Irwin and Barneby) and hemp sesbania, respectively, was evaluated [56]. The rain quantity and timing of the dew application caused differences in the severity of the disease and weed control, which were more critical for *C. truncatum* than for *A. cassia* after these bioherbicides were applied to their weed targets. We have not studied the overall effects of the dew timing and duration or of rainfall amounts and timing after AV application on horseweed, although this would be important for future studies.

Due to the concern of injury to non-target plants or crops, the specificity or selectivity of a bioherbicide is also an important consideration. The first reported plant host survey with AV on a variety of mono- and dicotyledonous plants demonstrated mortality levels >85% for several plants, including radish (*Raphanis sativa* L.), table beet (*Beta vulgaris* L.), chenopodium (*Chenopodium amaranticolor*), English pea (*Pisium sativum* L.), sicklepod, hemp sesbania, and jimsonweed (*Datura stramonium* L.) [41]. Severe reductions in dry-weight accumulation occurred in some other plant species. These results of AV, coupled with some in-depth studies on its bioherbicidal potential against some major weeds [45,46,47,48] in our laboratory, indicate a broad host range. The property of a broad weed control spectrum is advantageous for a bioherbicide, as well as for traditional herbicides. Conversely, of the 14 monocots tested, none exhibited mortality and the dry-weight reductions were low [41]. Another isolate of *Myrothecium* spp. from leafy spurge (*Euphorbia esula* L.) possessed a different host range when tested on several weeds [57,58,59]. Some *Myrothecium* spp. have been shown to be pathogenic to several ornamental and crop plants [57,60,61,62], and virulence and host range variations of different *Myrothecium* isolates have been reported [58,59,60,62]. Overall, these closely related microorganisms (AV and *Myrothecium* spp.) have bioherbicidal activity on several diverse plant species. When a bioherbicide becomes commercially available, usage recommendations or restrictions will be included on the label, analogous to the labeling for commercial herbicides. This will promote the safe use and application of the product.

Another important consideration, as demonstrated in some previous studies with AV and some other bioherbicides, is additive or synergistic pathogen–herbicide interactions to improve efficacy. We have previously reported additive or synergistic effects for weed control efficacy when the bioherbicides *Colletrotrichum truncatum* and AV were combined with glyphosate [63,64,65,66]. Other laboratories reported that the fungus *Pyricularia setariae,* applied to the weed green foxtail (*Setaria viridis*), was synergized by quinclorac, glufosinate, or glyphosate [67]. Combinations of AV and quinclorac caused additive or synergistic effects on growth, chlorophyll accumulation, and mortality in tissues of hemp sesbania, sicklepod, and kudzu [68]. AV has also been shown to have synergistic interactions with glyphosate on glyphosate-resistant Palmer amaranth plants [69], although some commercial glyphosate formulations can be antagonistic to AV [70].

## 5. Conclusions

In these studies, glyphosate-resistant and -susceptible *C. canadensis* plants were equally controlled by this AV isolate under greenhouse and field conditions. Further research will be required to establish and optimize other parameters, and to understand and to further characterize AV’s bioherbicidal activity on this weed under field conditions. In addition to interactions with glyphosate, synergistic interactions with other chemicals (herbicides, plant growth regulators, etc.) might be found to enhance AV’s efficacy. More research to improve the efficacy of this bioherbicide candidate is in progress.

Recently, the resistance mechanism in glyphosate-resistant horseweed was attributed to the rapid vacuolar sequestration of glyphosate via a tonoplast transporter [71]. Other researchers studying glyphosate-resistant horseweed imply the involvement of transporter genes and suggest that non-chemical control methods might be utilized to manage the spread of resistance of this problematic weed [72]. AV is a bioherbicide that has potential for the non-chemical management of some herbicide-resistant weeds such as *C. canadensis.*

## Data Availability

Not applicable.
